# Risk of Bleeding Associated With Ibrutinib in Patients With B-Cell Malignancies: A Systematic Review and Meta-Analysis of Randomized Controlled Trials

**DOI:** 10.3389/fphar.2020.580622

**Published:** 2020-11-20

**Authors:** Jinjin Wang, Ailin Zhao, Hui Zhou, Jinbing Zhu, Ting Niu

**Affiliations:** Department of Hematology, Institute of Hematology, West China Hospital, Sichuan University, Chengdu, China

**Keywords:** ibrutinib, B-cell malignancies, major bleeding, randomized controlled trials, overall bleeding

## Abstract

**Background:** Ibrutinib is an oral covalent Bruton’s tyrosine kinase inhibitor that has been approved for chronic lymphocytic leukemia (CLL)/small lymphocytic leukemia and some other B-cell malignancies. Some studies have found an increased risk of bleeding with ibrutinib. Some studies, however, found no significant differences in the risk of major bleeding between patients treated with ibrutinib and those with other regimens. So, a systematic review and meta-analysis of randomized controlled trials (RCTs) were performed to estimate the risk of bleeding associated with ibrutinib in patients with B-cell malignancies.

**Methods:** A systematic search of PUBMED, EMBASE, Central Register of Controlled Trials, and ClinicalTrials.gov was conducted from January 2000 to February 2020 to identify RCTs by comparing ibrutinib with other agents or placebo in B-cell malignancies. The RevMan software (version 5.3) was used to carry out this analysis, and the analyzed data were represented by risk ratios (RR) and 95% confidence intervals (CI).

**Results:** There were 11 eligible RCTs (4,288 patients). All studies reported major bleeding, and seven studies reported overall bleeding (any-grade bleeding). Ibrutinib was associated with a significantly increased risk of bleeding (overall bleeding and major bleeding) in patients with B-cell malignancies [RR = 2.56, 95% CI 1.68–3.90, *p* < 0.0001 and RR = 2.08, 95% CI 1.36–3.16, *p* = 0.0006, respectively]. The bleeding (overall bleeding and major bleeding) risk in patients with CLL was more obvious [RR = 3.08, 95% CI 2.07–4.58, *p* < 0.00001 and RR = 2.46, 95% CI 1.37–4.41, *p* = 0.003, respectively]. There were no statistically significant differences for risk of bleeding between the subgroups based on dose and treatment setting.

**Conclusion:** Ibrutinib was associated with a significantly higher risk of bleeding (both overall bleeding and major bleeding) in patients with B-cell malignancies, especially in CLL.

## Introduction

Ibrutinib is an orally administered covalent Bruton’s tyrosine kinase (BTK) inhibitor that has been approved for a variety of B-cell malignancies and graft-versus-host disease by US Food and Drug Administration (FOOD＆;Drug, 2017). The B-cell receptor (BCR) signaling pathway plays a vital role in the pathogenesis of several B-cell malignancies, including chronic lymphocytic leukemia (CLL), diffuse large B-cell lymphoma (DLBCL), and mantle-cell lymphoma (MCL) (Stevenson and Caligaris-Cappio, 2004; Gururajan et al., 2006; Merolle et al., 2018). BTK counts for much in the BCR signaling pathway, involved in various regulations of both normal and malignant B cells (Niiro and Clark, 2002; Chiorazzi and Ferrarini, 2003). Ibrutinib has shown beneficial effects, including a better progression-free survival, overall survival, and acceptable side-effect profiles (O'Brien et al., 2018a). Meanwhile, adverse reactions of ibrutinib have been found, mainly including diarrhea, fatigue, and nausea (O'Brien et al., 2018b). Fortunately, most patients can tolerate these adverse reactions and receive lasting treatment (Burger et al., 2015; Dreyling et al., 2016). An increasing number of studies are being conducted about ibrutinib in hematologic malignancies and solid tumors, and the indications for ibrutinib treatment may be more extensive in the future.

Although ibrutinib has a significant effect on hematologic conditions, early clinical trials reported increased incidence of bleeding in patients on ibrutinib compared with control therapies (Byrd et al., 2013; Wang et al., 2013). Despite the fact that some trials excluded patients on warfarin or vitamin K antagonists, bleeding events including major hemorrhage still occurred in the trials. The mechanism for bleeding events observed in ibrutinib clinical studies is not completely understood. Bleeding caused by ibrutinib is most likely attributable to platelet dysfunction. BTK plays a part in platelet signaling through GP1b and GPVI (a cell-surface receptor for collagen expressed on platelets), which mediate platelet accumulation and adhesion through von Willebrand factor and collagen, respectively. Ibrutinib inhibits platelet accumulation by inhibiting BTK, thereby increasing the risk of bleeding (Quek et al., 1998; Levade et al., 2014; Bye et al., 2015; Kamel et al., 2015; Lipsky et al., 2015; Alberelli et al., 2016).

Overall bleeding includes low-grade bleeding and major bleeding. Common low-grade bleeding includes subcutaneous or mucosal bleeding, contusion, nosebleed, and ecchymosis, which was often controlled by symptomatic support therapy or short-term withdrawal of ibrutinib (Shatzel et al., 2017). Major hemorrhage manifested itself as intracranial hemorrhage and gastrointestinal hemorrhage, which is evaluated by the Common Terminology Criteria for Adverse Events (CTCAE) in clinical practice (Institute, 2017). Some of the major hemorrhage can be controlled by discontinuing ibrutinib use and platelet infusion, but ibrutinib withdrawal may have a negative impact on the disease (Boriani et al., 2018). However, very severe bleeding, such as massive gastrointestinal bleeding and massive intracranial bleeding, can be fatal. Under such circumstances, some studies yet found no significant differences in the risk of major bleeding in patients treated with ibrutinib compared with other regimens (Yun et al., 2017; Brown et al., 2019).

Therefore, in order to clarify bleeding risk in ibrutinib treatment, a systematic review and meta-analysis of randomized controlled trials (RCTs) were performed to estimate the relative risk of overall bleeding (any grade bleeding) and major bleeding associated with ibrutinib in patients with B-cell malignancies, an attempt to provide convincing evidence for the clinical indications of ibrutinib.

## Methods

### Search Methods

A systematic search of PUBMED, EMBASE, Central Register of Controlled Trials (CENTRAL), and ClinicalTrials.gov was conducted from January 2000 to February 2020, by focusing on the following key words: Ibrutinib or Imbruvica or PCI-32765, bleeding or hemorrhage, clinical trial. There were no restrictions on literature in terms of language or region for the research. In this study, we followed the PRISMA (Preferred Reporting Items for Systematic reviews and Meta-Analyses) guidelines to determine the risk of bleeding in patients with B-cell malignancies treated with ibrutinib in randomized clinical trials.

### Study Selection

All RCTs comparing ibrutinib with other agents or placebo in the treatment of patients with B-cell malignancies were considered to be eligible. We excluded the RCTs not mentioning bleeding as a therapy-related adverse event. Data on bleeding events were collected from the included studies. The primary outcome was overall bleeding and major bleeding. Overall bleeding was defined as bleeding events of all grades. According to the Common Terminology Criteria for Adverse Events (CTCAE) (Institute, 2017), any of the following items was considered as major bleeding: 1) any treatment-emergent hemorrhagic adverse events (AEs) of Grade 3 or higher, 2) any treatment-emergent serious AEs of bleeding of any grade, 3) any treatment-emergent central nervous system hemorrhage/hematoma of any grade, and 4) all hemorrhagic events requiring transfusion of red blood cells.

### Data Extraction and Assessment of Bias

The eligible studies were reviewed and data were independently extracted by two authors (JW and HZ), and any disagreement was resolved by the third author. Data were extracted according to a preliminarily designed Microsoft Excel spreadsheet. Our research extracted first author, ClinicalTrials.gov number, disease, age, treatment, number of patients, median follow-up, and bleeding events. The risk of bias in the included studies was determined by using Cochrane’s Risk of Bias Tool for quality assessment of randomized controlled trials by two authors independently and cross-checked. The assessment contained seven domains: random sequence generation, allocation concealment, blinding of participants and personnel, blinding of outcome assessment, incomplete outcome data, selective reporting, and other bias.

### Statistical Analyses

Statistical analysis was performed using RevMan software version 5.3. Bleeding events in the included studies were pooled with the Mantel–Haenszel method to estimate the relative risk of bleeding. The Q statistic test and the I^2^ statistic test were used to assess heterogeneity. The I^2^ statistic ranges from 0 to 100% (I^2^ < 25%, low heterogeneity; I^2^ 25–50%, moderate heterogeneity; I^2^ > 50%, substantial heterogeneity). A random-effect model was used when potential heterogeneity existed (I^2^ > 50%); otherwise, the fixed-effect model was employed. The 95% confidence interval was estimated to assess the dispersion of the effect size in different settings. The funnel plot method was applied to publication bias assessment. Subgroup analysis was performed to solve heterogeneity. Sensitivity analysis was carried out to assess the stability of the pooled results.

## Results

### Study Characteristics

**FIGURE 1:**
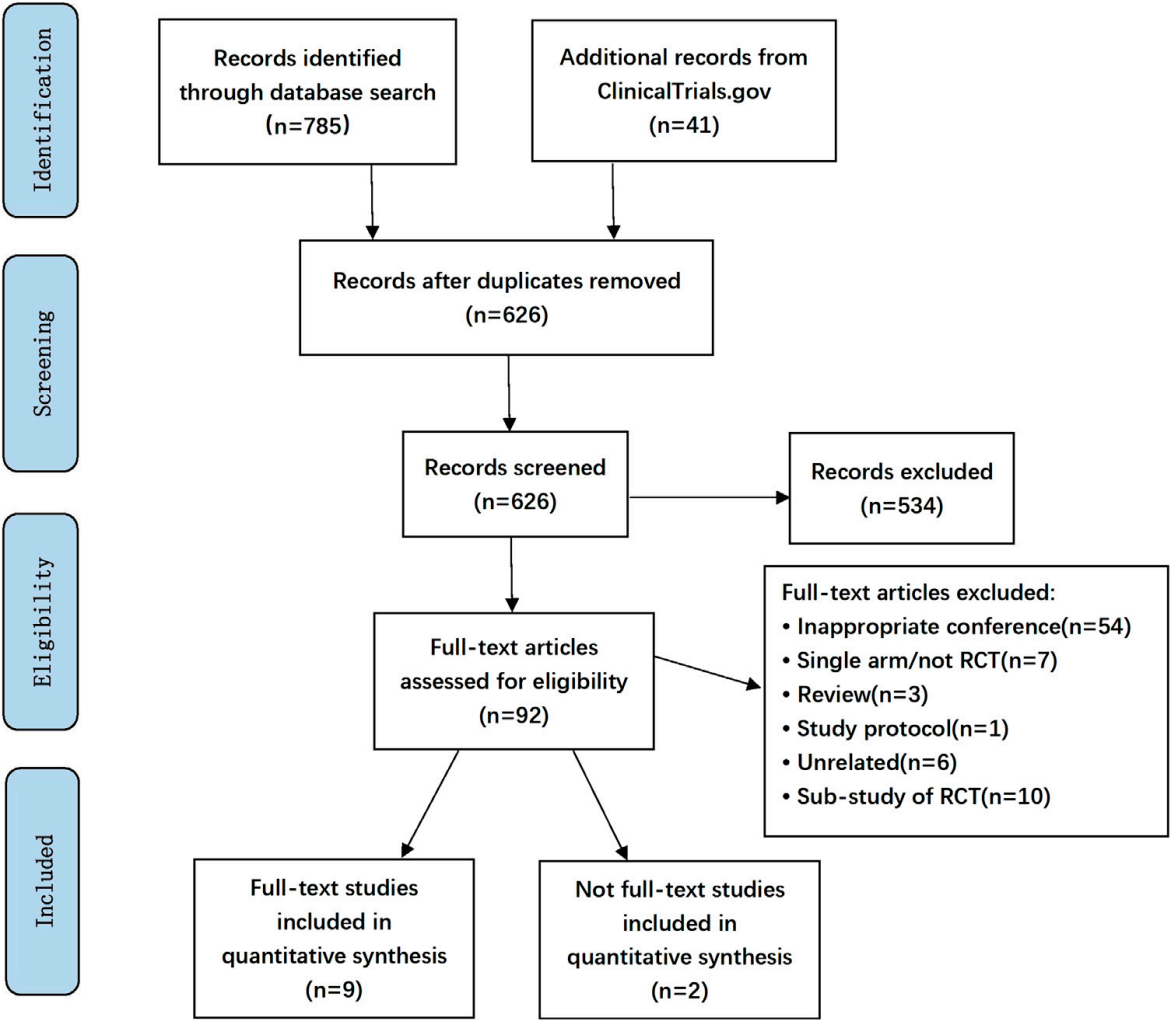
The flow chart.

The search strategy yielded 826 unique abstracts, among which 785 articles were from the database search and 41 studies were from the ClinicalTrials.gov. After screening abstracts and full-text analyses, 11 eligible RCTs were identified (Figure 1), of which nine were full-text articles (Byrd et al., 2014; Burger et al., 2015; Chanan-Khan et al., 2016; Dreyling et al., 2016; (Cramer et al., 2018; Dimopoulos et al., 2018; Huang et al., 2018; Woyach et al., 2018; Moreno et al., 2019; Shanafelt et al., 2019). The other two were a conference abstract (Langerbeins et al., 2019) and a trial registered in ClinicalTrials.gov, respectively (ClinicalTrials.gov, 2013). A total of 4,288 patients (2,357 patients in the ibrutinib arms and 1931 in the control arms) were included. Characteristics of all these trials enrolled in the analysis are summarized in Table 1. Median age of the participants varied from 56.7 to 73.0 years. Median follow-up time ranged from 9.4 to 38.7 months. In the 11 included studies, eight trials were conducted in patients with CLL, one was done in patients with Waldenstrom macroglobulinemia (WM), one in patients with MCL, and the other in DLBCL. Ibrutinib was compared to an active agent in seven trials, while it was compared to placebo in others. The daily dose of ibrutinib was 420 mg in nine studies, while it was used with a dose of 560 mg daily in patients with MCL or DLBCL in the other two trials. Major bleeding was reported in all studies, and overall bleeding was reported in seven studies. All trials excluded patients on warfarin or other vitamin K antagonists, except for the study that initially allowed but later banned the use of warfarin (or other vitamin K antagonists).

**TABLE 1:** Characteristics of studies included in meta-analysis.

Study	Clinic trials gov	Disease	Age(y), median (range)	Study arms	No. of patients (ibrutinib vs. placebo arm)	Ibrutinib daily dose (mg)	Median follow up (mo)	Over all bleeding incidence (ibrutinib/control)	Major bleeding incidence (ibrutinib/control)
Byrd (Byrd et al., 2014)	NCT01578707	RR CLL/SLL	67 (30–88)	Ibrutinib vs. Ofatumumab	391 (195 vs. 196)	420	9.4	86/24	2/3
Burger (Burger et al., 2015)	NCT01722487	Untreated CLL/SLL	73 (65–89)	Ibrutinib vs. Chlorambucil	267 (135 vs. 132)	420	18.4	N/R	6/3
Chanan-Khan (Chanan-Khan et al., 2016)	NCT01611090	RR CLL/SLL	64 (31–86)	Ibrutinib + Bendamustine + Rituximab vs. placebo + Bendamustine + Rituximab	574 (287 vs. 287)	420	17	89/42	11/5
Dreyling (Dreyling et al., 2016; Cramer et al., 2018)	NCT01646021	RR MCL	68 (IQR 13)	Ibrutinib vs. Temsirolimus	278 (139 vs. 139)	560	38.7	56/46	13/7
Dimopoulos (Dimopoulos et al., 2018)	NCT02165397	WM	69 (36–89)	Ibrutinib + Rituximab vs. Placebo + Rituximab	150 (75 vs. 75)	420	26.5	38/16	3/3
Huang (Huang et al., 2018)	NCT01973387	RR CLL/SLL	66 (21–87)	Ibrutinib vs. Rituximab	156 (104 vs. 52)	420	17.8	30/2	3/1
Woyach (Woyach et al., 2018)	NCT01886872	Untreated CLL	71 (65–89)	Ibrutinib vs. Ibrutinib + Rituximab vs. Bendamustine + Rituximab	537 (176 vs. 180 vs. 181)	420	38	N/R	8/0
Moreno (Moreno et al., 2019)	NCT02264574	Untreated CLL/SLL	71 (66–76)	Ibrutinib + Obinutuzumab vs. chlorambucil + Obinutuzumab	228 (113 vs. 115)	420	31.3	10/0	4/0
Shanafelt (Shanafelt et al., 2019)	NCT02048813	Untreated CLL/SLL	56.7 (49.3–64.1)	Ibrutinib + Rituximab vs. Fludarabine + Cyclophosphamide + Rituximab	510 (352 vs. 158)	420	33.6	N/R	4/0
(Deeks, 2017)	NCT01855750	Untreated DLBCL	62 (19–88)	Ibrutinib + R-CHOP vs. Placebo + R-CHOP	834 (416 vs. 418)	560	34.8	N/R	11/6
Langerbeins (Langerbeins et al., 2019)	NCT02863718	Untreated CLL	—	Ibrutinib vs. Placebo	363 (185 vs. 178)	420	31	51/17	6/2

N/R, not report; CTCAE, common terminology criteria for adverse events; R-CHOP, rituximab, cyclophosphamide, doxorubicin, vincristine, and prednisone; AE, adverse events; RR, relapse/refractory; CLL/SLL, chronic lymphocytic leukemia/Small lymphocytic leukemia; MCL, mantle-cell lymphoma; WM, waldenstrom macroglobulinemia; DLBCL, diffuse large B-cell lymphoma.

### Rate of Bleeding Across Studies

The overall bleeding events accounted for 32.8% in patients with B-cell malignancies receiving ibrutinib, compared with 14.1% in the control arm. While in B-cell malignancies, major bleeding was noted in 3.0% of patients in the ibrutinib arm, compared with 1.6% in the control arm. In eight RCTs on CLL, overall bleeding occurred in 30.1% of patients in the ibrutinib arm compared with 10.3% in the control arm, while major bleeding occurred in 2.5% of patients in the ibrutinib arm compared to 1.1% in the control arm. In studies where ibrutinib was used as the first-line therapy, overall bleeding was noted in 20.5% of patients receiving ibrutinib compared with 5.8% in the control group. In studies where ibrutinib was used in relapsed/refractory settings, overall bleeding was noted in 36.0% of patients in the ibrutinib group vs. 16.9% in the control group. Rates of bleeding events are summarized in Tables 2, 3.

**TABLE 2:** Rate of overall bleeding across studies.

	Ibrutinib	Control
B-cell malignancies (%)	360/1,098 (32.8%)	147/1,402 (14.1%)
CLL (%)	266/884 (30.1%)	85/828 (10.3%)
First-line (%)	61/298 (20.5%)	17/293 (5.8%)
Relapsed/refractory (%)	261/725 (36.0%)	114,674 (16.9%)

The percentage (%) is calculated from the events/total number of patients. CLL, chronic lymphocytic leukemia.

**TABLE 3:** Major bleeding across studies.

	Ibrutinib	Control
B-cell malignancies (%)	71/2,357 (3.0%)	30/1931 (1.6%)
CLL (%)	44/1727 (2.5%)	14/1,299 (1.1%)
First-line (%)	39/1,577 (2.5%)	11/1,182 (0.9%)
Relapsed/refractory (%)	29/725 (4.0%)	16/674 (2.4%)

The percentage (%) is calculated from the events/total number of patients. CLL, chronic lymphocytic leukemia.

### Risk of Bleeding in B-Cell Malignancies

**FIGURE 2:**
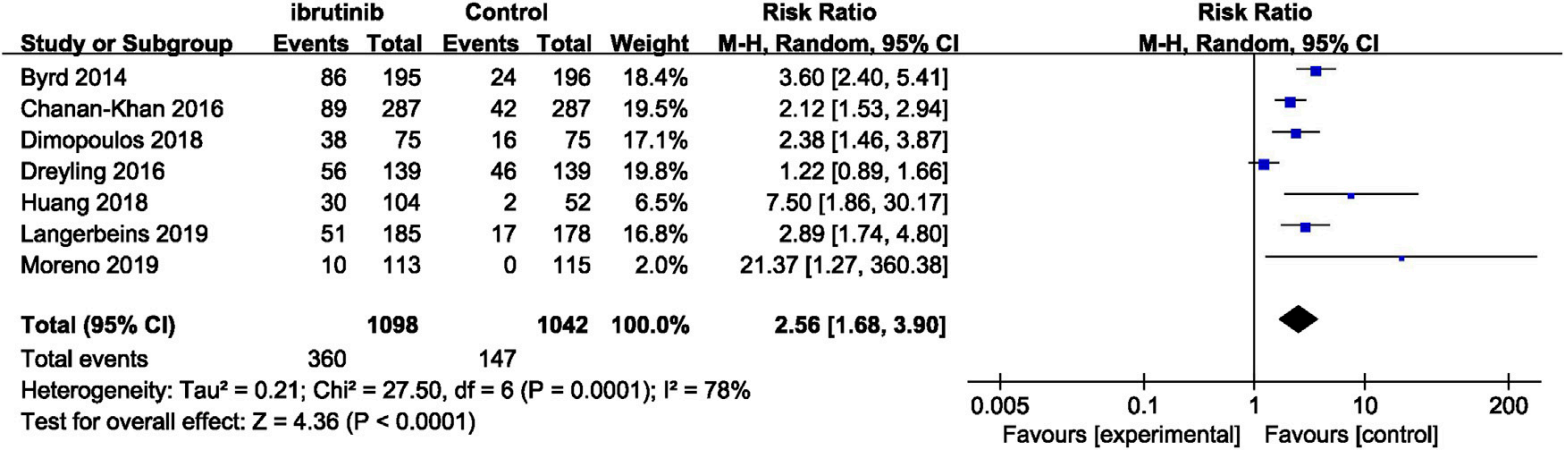
Forest plot of relative risk of overall bleeding in B-cell malignancies.

**FIGURE 3:**
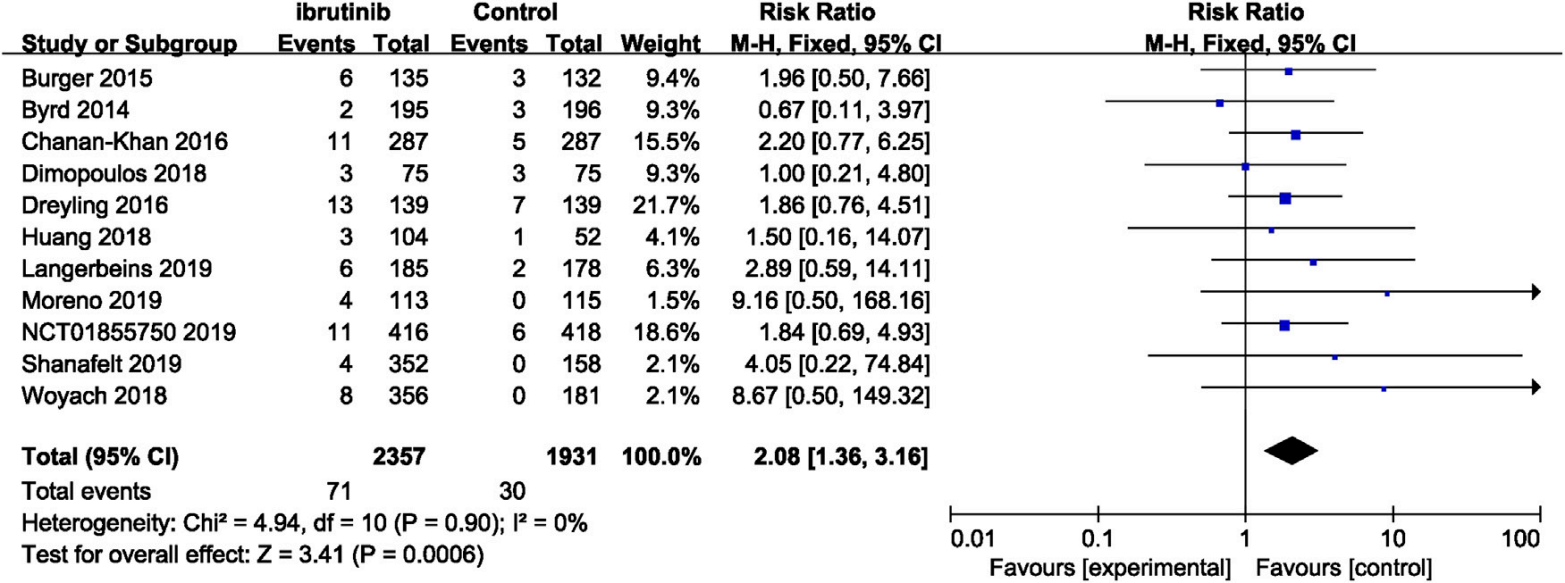
Forest plot of relative risk of major bleeding with in B-cell malignancies.

Seven studies reported overall bleeding. Compared with control treatments, ibrutinib treatment was associated with a statistically significant increased risk of overall bleeding in patients with B-cell malignancies (RR = 2.56, 95% CI 1.68–3.90, *p* < 0.0001, I^2^ = 78%; Figure 2). The random-effect model was used because of the significant heterogeneity of studies. Of the 11 studies, nine studies showed a significantly increased risk of major bleeding in ibrutinib group, one study showed no significant difference between the ibrutinib group and the control group, while the other study showed an increased risk of major bleeding in the control group. Overall, the pooled estimate showed that ibrutinib increased the risk of major bleeding (RR = 2.08, 95% CI 1.36–3.16, *p* = 0.0006, I^2^ = 0%; Figure 3) by using a fixed-effect model.

### Risk of Bleeding in Chronic Lymphocytic Leukemia

**FIGURE 4:**
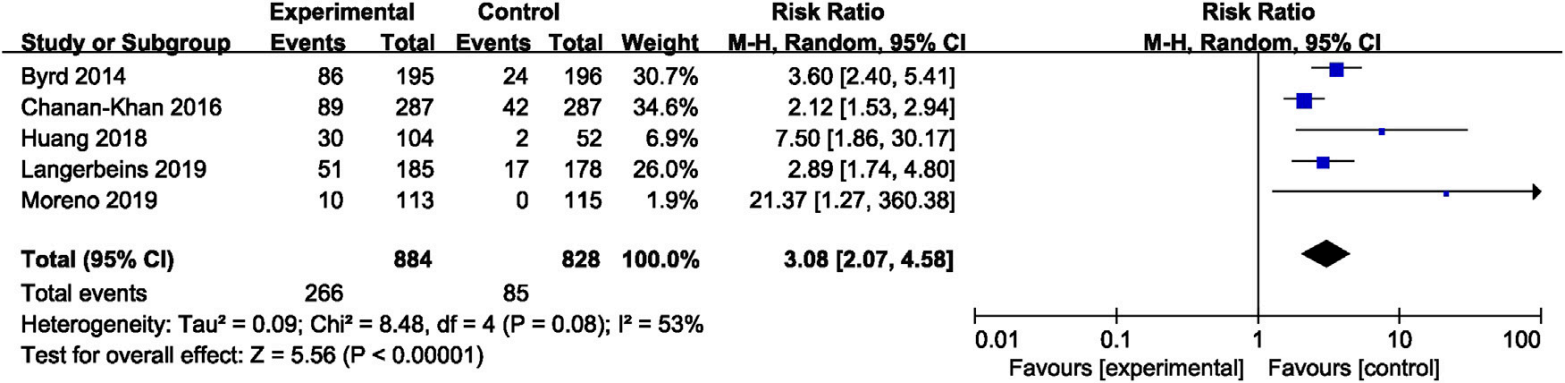
Forest plot of relative risk of overall bleeding in CLL.

**FIGURE 5:**
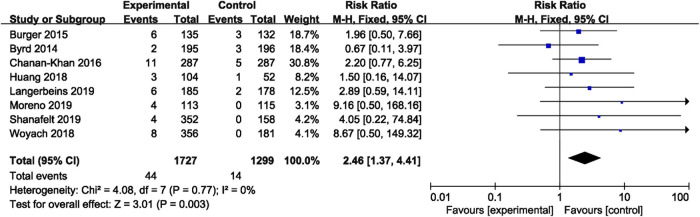
Forest plot of relative risk of major bleeding in CLL.

In CLL, the pooled risk ratio in five studies showed a more than three-fold increase in the risk of overall bleeding with ibrutinib (RR = 3.08, 95% CI, 2.07–4.58, *p* < 0.00001, I^2^ = 53%; Figure 4). The random-effect model was used because of heterogeneity. The risk of major bleeding was found to be significantly higher in patients of CLL with ibrutinib than that in the control group (RR = 2.46, 95% CI, 1.37–4.41, *p* = 0.003, I^2^ = 0; Figure 5) through the fixed-effect model.

### Subgroup Analysis

**FIGURE 6:**
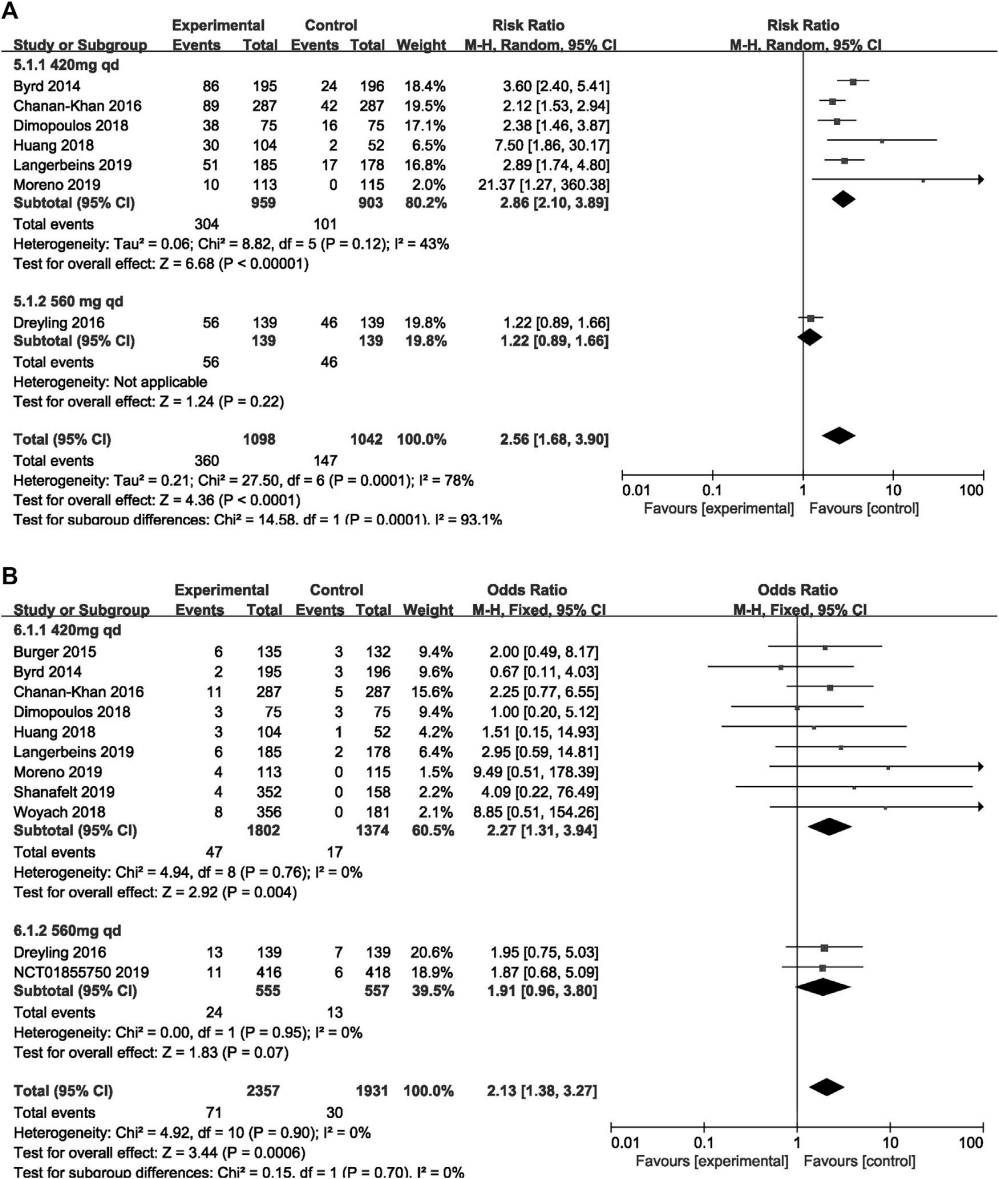
Forest plot of relative risk of overall **(A)** and major **(B)** bleeding in different dosage of ibrutinib.

The subgroup analysis was performed among patients with different dosage and treatment settings. For patients on ibrutinib with a dosage of 420 mg/day, the risk of overall bleeding was significantly higher than that in the control group (RR = 2.86, 95% CI 2.10–3.89, *p* < 0.00001). For those on ibrutinib with a dosage of 560 mg/day, the difference was not significant (RR = 1.22, 95% CI 0.89–1.66, *p* = 0.22; Figure 6A). In terms of major bleeding, patients who received ibrutinib treatment with a dosage of 420 mg/day encountered significantly elevated risk of major bleeding compared to the control group (RR = 2.27, 95% CI 1.31–3.94, *p* = 0.004). No significant difference in major bleeding was found between the ibrutinib group when the dosage was 560 mg/day and control group (RR = 1.91, 95% CI 0.96–3.80, *p* = 0.07; Figure 6B).

**FIGURE 7:**
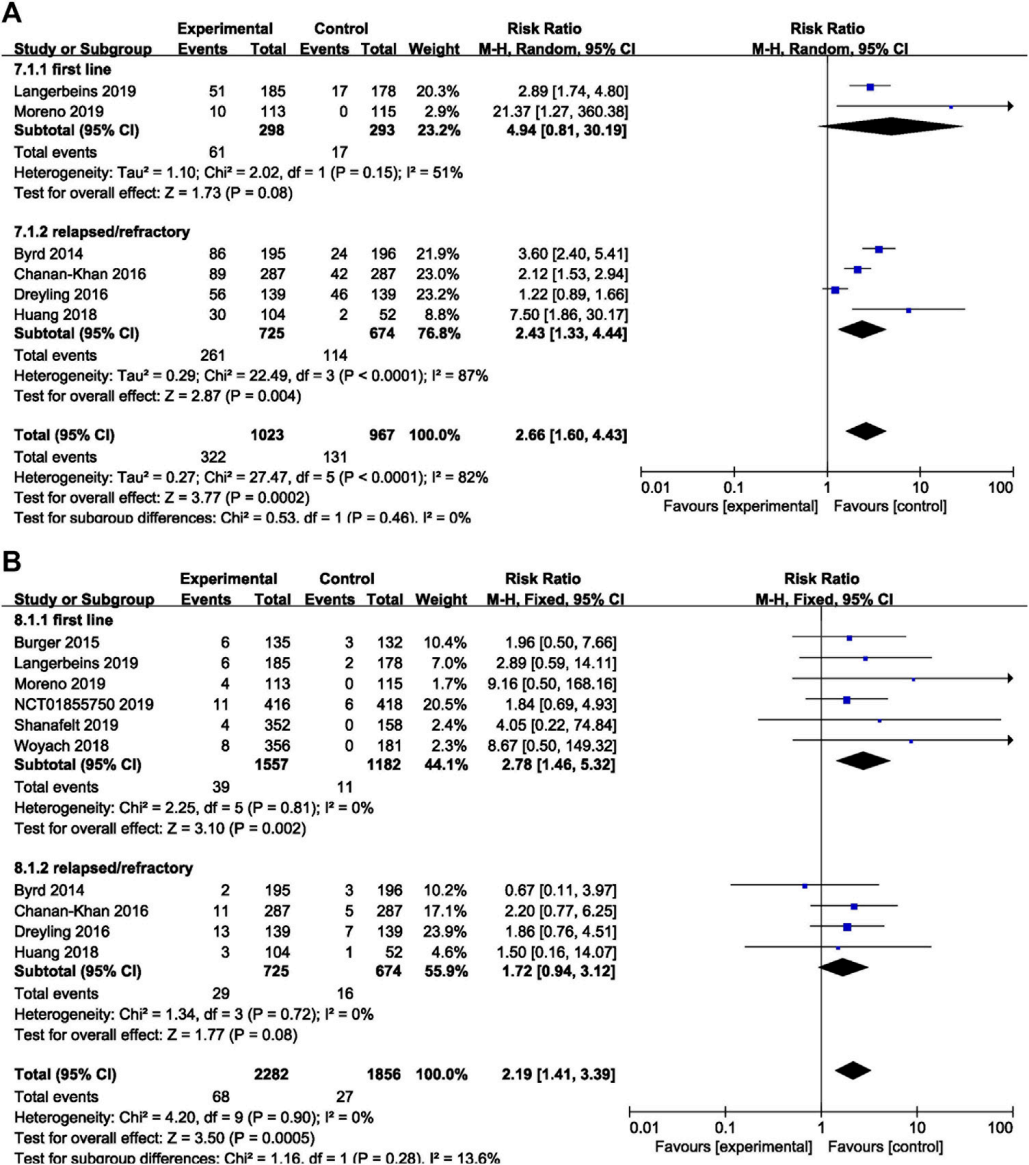
Forest plot of relative risk of overall **(A)** and major bleeding **(B)** in different treatment setting.

In terms of overall bleeding, treatment-naïve patients on ibrutinib tended to experience more overall bleeding events (RR = 4.94, 95% CI 0.81–30.19, *p* = 0.08) than the control group, although the difference was not significant. Refractory/relapsed patients who received ibrutinib treatment had a significantly increased risk of overall bleeding compared to control group (RR = 2.43, 95% CI 1.33–4.44, *p* = 0.004; Figure 7A). Regarding major bleeding, treatment-naïve patients who received ibrutinib treatment experienced significantly more major bleeding events than the control group (RR = 2.78, 95% CI 1.46–5.32, *p* = 0.002). But no significant difference in major bleeding in refractory/relapsed patients was identified between the ibrutinib group and control group (RR = 1.72, 95% CI 0.94–3.12, *p* = 0.08; Figure 7B).

### Study Heterogeneity and Publication Bias

**FIGURE 8:**
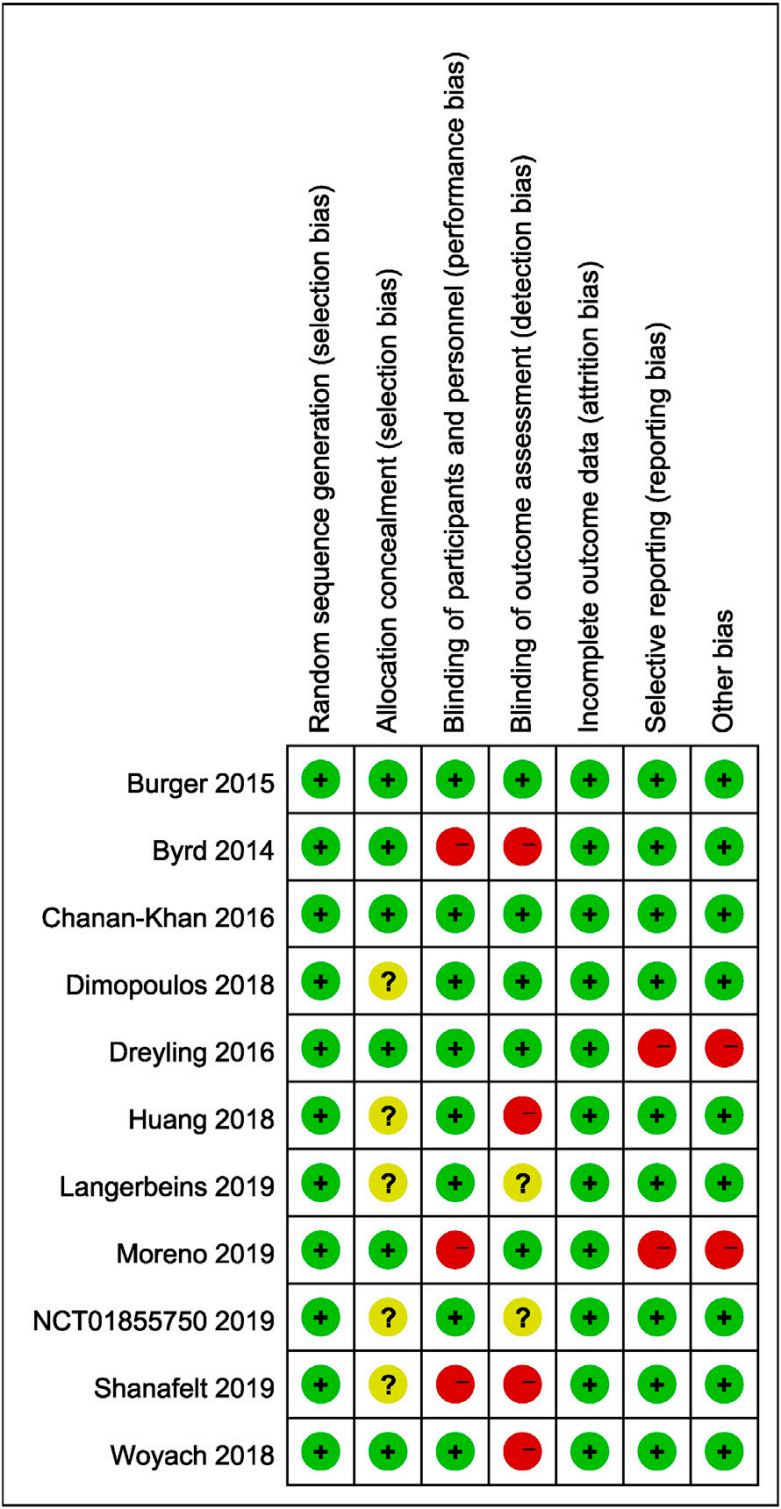
Risk of bias plot of included studies.

Two studies without full texts cannot be evaluated comprehensively. Five included studies were open-labeled, and six studies were double blind. The baseline demographic characteristics in each study were well balanced between experimental and control arms. The quality evaluation is shown in Figure 8. The results of our research remained unchanged after sensitivity analysis. No publication bias was noted when assessed by funnel plot.

## Discussion

Our results suggested that patients treated with ibrutinib had a significantly increased risk of bleeding (both overall bleeding and major bleeding) compared with patients receiving alternative therapies or placebo. Especially in CLL, the risk of bleeding in patients with ibrutinib was even more robust. In our analysis, although the forest plot (Figure 6) showed a difference in the risk of overall bleeding caused by the two doses of 420 and 560 mg, there was only one study in the 560 mg group, so the analysis results were not of practical clinical significance and of little value. Therefore, the increased risk of overall and major bleeding in the ibrutinib group was consistent among different dosages and disease settings in subgroup analyses.

The United States Food and Drug Administration has been extending the approved indications for ibrutinib in various lymphoid malignancies, including CLL, marginal zone lymphoma, and WM, since its first approval for mantle-cell lymphoma in 2013. An international, observational, retrospective analysis (the largest series-reported to date on ibrutinib in a real-world setting) showed that ibrutinib was effective and well tolerated in clinical practice (Hillmen et al., 2018). The role of ibrutinib in B-cell malignancies is indisputable, as it improves progression-free survival and overall survival (Deeks, 2017). Although ibrutinib was well-tolerated, the incidence of bleeding was increased, including major bleeding complications (subdural hematomas, gastrointestinal bleeding, and hematuria) in patients with ibrutinib (Byrd et al., 2013; Dreyling et al., 2016). Low-grade bleeding, such as subcutaneous or mucosal bleeding, can be resolved by symptomatic support therapy (Shatzel et al., 2017). However, serious or grade 3 or higher hemorrhages often lead to sequelae, such as hemiplegia or limb dysfunction due to intracranial hemorrhage. Very severe bleeding is the direct cause of death. An increasing number of meta-analyses have shown that ibrutinib increases the risk of low-grade hemorrhage. However, most of the literature suggests that ibrutinib does not increase the risk of major bleeding (Caron et al., 2017; Yun et al., 2017). In a real-world retrospective trial involving a total of 95 patients receiving ibrutinib monotherapy, 46% of patients had low-grade bleeding events without any major bleeding (Winqvist et al., 2016). In our meta-analysis, further evidence suggests that ibrutinib significantly increases the risk of overall bleeding. Some studies found no significant difference in risk of major bleeding between the ibrutinib group and alternative treatment group, but our analysis identified a significantly increased risk of major bleeding. The reason why our conclusion differed from those of previous studies could be that although the incidence of major bleeding caused by ibrutinib was low, the studies included in our analysis were randomized controlled trials with larger sample sizes (4,288 patients)and longer follow-up, which made it more likely to detect major bleeding events.

In our analysis, the bleeding risk was increased more significantly in the ibrutinib group with CLL/SLL. In CLL, the overall bleeding risk of ibrutinib was three times higher than that of the control group, and the risk of major bleeding was also increased. This finding could be related to a defect in platelet accumulation in CLL patients, which was likely exacerbated by ibrutinib, and the risk of bleeding might increase (Lipsky et al., 2015; Shatzel et al., 2017). A previous meta-analysis did not clearly indicate that the bleeding risk after receiving ibrutinib was increased more significantly in CLL patients. Therefore, it is necessary to pay special attention to the bleeding events of CLL patients when using ibrutinib in clinical practice.

For the low-grade bleeding, such as skin or mucosal bleeding and nosebleed, it can be controlled by the use of hemostatic drugs and other symptomatic treatment (Shatzel et al., 2017). For severe hemorrhage, infusion of platelets regardless of platelet count is recommended, and antifibrinolytic drugs may be used if bleeding persists after platelet infusion (Boriani et al., 2018; Stephens and Byrd, 2019). Some patients even need to stop using ibrutinib permanently. Surgical intervention is required when necessary. The phase III study, however, showed that platelet transfusion was not recommended for central nervous system (CNS) hemorrhage caused by ibrutinib (Baharoglu et al., 2016). But more research is required to confirm the risk/benefit of transfusion in the setting of ibrutinib-associated CNS bleeding. Therefore, the decision to transfuse platelets in this setting should be individualized in clinical practice. For patients undergoing surgery or invasive operation, it is recommended to stop ibrutinib, but the interruption of the drug may have a negative effect on B-cell malignancies. Therefore, whether the patient should stop ibrutinib treatment needs to be evaluated individually according to the type of surgery and the actual bleeding risk it may cause.

Many patients receiving ibrutinib have a history of cardiovascular disease or risk factors that require anticoagulant (AC)/antiplatelet (AP) therapy for primary or secondary prevention. Whether patients taking ibrutinib can take these drugs at the same time is controversial. In our analysis, 10 RCTs excluded patients receiving warfarin or other vitamin K antagonists because of their increased risk of bleeding, but the risk of bleeding was still significantly increased. This suggests that ibrutinib does increase the risk of bleeding. AC or AP therapy is known to be critical for patients with atrial fibrillation, blood clots, or coronary stent implantation. However, it remains to be solved whether ibrutinib can be used for patients with B-cell malignancies in combination with AC or AP agents. In a multi-center, open-label phase II registration trial, where the concomitant use of warfarin/other vitamin K antagonist was allowed initially, subdural hematomas were reported in 4/111 patients (grade 3 in two patients). All four patients received aspirin/warfarin within 2 days of the event (Wang et al., 2015). Another single-center study of 71 patients taking ibrutinib, most of whom took AC or AP drugs at the same time, showed that 18% of patients had major bleeding (Kunk et al., 2016). These studies, to some extent, suggested that AC/AP therapy may further increase the risk of bleeding. A retrospective study showed that the use of ibrutinib in standard clinical settings increased the risk of major bleeding at a higher rate than previously reported, because in the real-world study, some patients had anemia or an increased international normalized ratio or needed anticoagulant and/or antiplatelet drugs, which greatly increased the risk of bleeding during treatment of ibrutinib (Mock et al., 2018). Meanwhile, patients with CLL might show some degree of platelet accumulation defects (impaired response to collagen or ADP agonists) compared with healthy controls, which could be exacerbated by ibrutinib and resulted in increased risk of bleeding (Lipsky et al., 2015). Consequently, patients who use ibrutinib in an actual clinical setting may have a higher risk of bleeding from low-grade bleeding to severe bleeding. Until more reliable data can be used to determine risk factors of bleeding, the decision to begin AC/AP therapy should be individualized, considering available evidence on the benefits and risks of treatment. In fact, some patients have to use anticoagulants because of their conditions such as venous thromboembolism or atrial fibrillation. Studies are beginning to explore the safety of direct oral anticoagulants (DOACs) such as dabigatran, rivaroxaban, and apixaban in patients with ibrutinib. In a recent retrospective study, although the risk of bleeding was still high when ibrutinib was used in combination with DOACs, most of the bleeding was grade 1–2, so the safety profile of concurrent treatment of both DOACs and ibrutinib seemed tolerable (Raz et al., 2020). However, data were limited, and this study was a retrospective study with a small sample size, so more studies with larger sample size are needed to explore the safety of ibrutinib combined with DOACs.

In addition to ibrutinib, there are more second-generation BTK inhibitors. Acalabrutinib is another “second-generation” BTK inhibitor with increased selectivity for BTK. Early phase I and II trials showed no major hemorrhage (Byrd et al., 2016). Many research works on acalabrutinib are ongoing (ClinicalTrials.gov, 2015; ClinicalTrials.gov, 2019). Zanubrutinib is also a potential and highly selective inhibitor of BTK. A multi-center phase I study showed that 8.5 and 16.0% of the 94 patients with CLL/SLL who received zanubrutinib concomitantly accepted AC or AP therapy, respectively, and only one developed major bleeding (Tam et al., 2019). This suggests that zanubrutinib may have a lower incidence of bleeding, but more reliable data are needed.

Several limitations in our analysis should be mentioned. First, we excluded several studies which did not mention bleeding, possibly due to the low incidence of ibrutinib-associated bleeding. These studies possibly paid little attention to this rare event when designing the trial. This might bring potential bias to our study. Second, although all studies excluded patients who also received warfarin or vitamin K antagonists, there were no restrictions on other anticoagulants or antiplatelet agents. This might affect the final result. Third, the duration of drug exposure was different between the experimental and control groups, which might influence the final results. Finally, the follow-up time was different among studies, which could also affect the observance of bleeding events. Meanwhile the number of included studies is small, more studies with larger sample size are required to confirm our results.

## Conclusion

In conclusion, our meta-analysis demonstrated that ibrutinib was associated with a significantly higher risk of bleeding (both overall bleeding and major bleeding) in patients with B-cell malignancies, especially in CLL. This analysis provides stronger evidence for the close monitoring of bleeding in patients receiving ibrutinib. However, the mechanisms by which ibrutinib increases bleeding, the risk factors for bleeding, and feasibility of simultaneous application of anticoagulant therapy require further investigation.

## Author Contributions

JW collected, analyzed the data and wrote the article. JW and HZ performed the statistical analysis. JW and JZ prepared the pictures and tables. TN provided the idea and modified the article. AZ analyzed and interpreted the data, and participated in the revision of the article. All authors read and approved the final manuscript.

## Conflict of Interest

The authors declare that the research was conducted in the absence of any commercial or financial relationships that could be construed as a potential conflict of interest.
